# Evaluation of blood-brain barrier function by quotient alpha2 macroglobulin and its relationship with interleukin-6 and complement component 3 levels in neuropsychiatric systemic lupus erythematosus

**DOI:** 10.1371/journal.pone.0186414

**Published:** 2017-10-16

**Authors:** Tomoyuki Asano, Hiromi Ito, Yoshinobu Kariya, Kyoka Hoshi, Akioh Yoshihara, Yoshikazu Ugawa, Hideharu Sekine, Shunsei Hirohata, Yoshiki Yamaguchi, Shuzo Sato, Hiroko Kobayashi, Kiyoshi Migita, Hiromasa Ohira, Yasuhiro Hashimoto, Hiroshi Watanabe

**Affiliations:** 1 Department of Rheumatology, School of Medicine, Fukushima Medical University, Fukushima, Japan; 2 Department of Biochemistry, School of Medicine, Fukushima Medical University, Fukushima, Japan; 3 Department of Neurology, School of Medicine, Fukushima Medical University, Fukushima, Japan; 4 Department of Immunology, School of Medicine, Fukushima Medical University, Fukushima, Japan; 5 Department of Rheumatology and Infectious Diseases, Kitasato University School of Medicine, Kanagawa, Japan; 6 Structural Glycobiology Team, Systems Glycobiology Research Group, RIKEN-Max Planck Joint Research Center, RIKEN Global Research Cluster, Wako, Saitama, Japan; 7 Department of Gastroenterology, School of Medicine, Fukushima Medical University, Fukushima, Japan; Peking University First Hospital, CHINA

## Abstract

Although quotient of alpha2 macroglobulin (Qα2MG) was previously reported to be useful for the evaluation of blood–brain barrier (BBB) function, it is not commonly used. We therefore evaluated BBB function among the various subsets of neuropsychiatric systemic lupus erythematosus (NPSLE) using quotient Q α2MG. Furthermore, we determined the correlation between Q α2MG and cerebrospinal (CSF) interleukin (IL)-6 level and quotient complement component 3 (Q C3). To determine intrathecal production of C3, the C3 index (Q C3/Q α2MG) was also calculated. Fifty-six patients with SLE were included in this study. Of these, 48 were diagnosed with NPSLE, consisting of 30 diffuse NPSLE patients (acute confusional state (ACS): n = 14, non-ACS: n = 16) and 18 patients with focal NPSLE. CSF IL-6 concentration, and paired serum and CSF levels of α2MG and C3, were measured by enzyme-linked immuno solvent assay (ELISA). The Q α2MG, Q C3, and C3 index were then calculated. Q α2MG, Q C3, and IL-6 concentrations in the CSF were significantly elevated in NPSLE compared with non-NPSLE. Among the subsets of NPSLE, significant increases in Q α2MG, CSF IL-6, and Q C3 were observed in ACS compared with non-ACS or focal NPSLE. There was a positive correlation between CSF IL-6 level and Q α2MG, as well as between Q C3 and Q α2MG, in diffuse NPSLE. There were no significant differences in C3 index between NPSLE and non-NPSLE, as well as among the subgroups of NPSLE. Our study suggests that BBB disruption is present in ACS, and elevated levels of IL-6 and C3 in CSF in diffuse NPSLE, especially in ACS, might result from their entry to the CSF from the systemic circulation through the damaged BBB, as well as increased intrathecal production. Furthermore, Q α2MG might be useful for the evaluation of BBB integrity.

## Introduction

Systemic lupus erythematosus (SLE) is an inflammatory autoimmune disease characterized by the production of autoantibodies and the formation of immune complexes. Deposition of immune complexes triggers the classical complement pathway, resulting in inflammation and tissue injury [1]. Neuropsychiatric manifestations have been reported to occur in 14% to over 80% of adult SLE patients [2, 3]. Although neuropsychiatric SLE (NPSLE) is one of the major causes of death in SLE patients, its pathogenic mechanisms remain to be elucidated.

In 1999, the American College of Rheumatology (ACR) classified the clinical features of NPSLE in 19 specific neuropsychiatric syndromes, which can be segregated into two main syndromes: central nervous system (CNS) syndromes and peripheral nervous system syndromes. CNS syndromes are further divided into diffuse syndromes (diffuse NPSLE; acute confusional state (ACS), anxiety disorder, cognitive dysfunction, mood disorder, psychosis) and focal syndromes (focal NPSLE; cerebrovascular disease, demyelinating syndrome, headache, movement disorder, seizure disorder), mostly depending upon the anatomical sites of the pathology in the CNS [4–6].

Multiple mechanisms are involved in the pathogenesis of NPSLE including autoantibody-mediated neurotoxicity, direct action of inflammatory cytokines, microvasculopathy (which may be attributed to complement activation), thrombotic vasculopathy caused by anti-phospholipid antibodies (aPL), intrathecal production of immune complexes, blood–brain barrier (BBB) disruption, and accelerated atherosclerosis [7–9]. Generally, focal neuropsychiatric manifestations are due to thrombotic vasculopathy mediated by aPL and immune complexes. In contrast, diffuse neuropsychiatric manifestations reflect neurotoxicity mediated by autoantibodies or inflammation with increased inflammatory mediators.

The BBB is a selective semipermeable membrane that separates the circulatory system from the brain. The BBB, formed from polarized brain endothelial cells that are connected by tight intracellular junctions, protects the brain microenvironment. BBB disruption occurs in several pathologies such as stroke, infection, and autoimmune disorders including NPSLE [10, 11]. The CSF/serum quotient of albumin, known as quotient albumin (Q albumin), is widely accepted as a biomarker for estimating BBB function, because albumin is solely produced in the liver [12]. Recent studies demonstrated that Q albumin was increased in NPSLE, especially in ACS diffuse NPSLE, which raises the possibility that pathogenic autoantibodies such as anti-NR2 and anti-Sm may enter the CSF from the systemic circulation through a disrupted BBB, and play a critical role in the pathogenesis of NPSLE [13–15].

Interleukin-6 (IL-6) is an inflammatory mediator that contributes to the pathogenesis of NPSLE. Recent studies demonstrated that IL-6 is increased in the CSF of patients with NPSLE, especially those with ACS [16–18], and is a clinically useful marker for lupus psychosis [19]. However, in those studies, IL-6 levels in CSF were only compared between NPSLE and non-NPSLE [16, 17], or ACS diffuse NPSLE and NPSLE other than ACS (non-ACS diffuse NPSLE + focal NPSLE) [18]. To the best of our knowledge, there have been few reports regarding the independent measurement of CSF IL-6 levels in non-ACS diffuse NPSLE or focal NPSLE.

Complement activation also contributes to the pathogenesis of NPSLE. Reports of brain histopathology showed that microvasculopathy, which may be due to aPL and activation of complement, was the most common finding in patients with NPSLE [7]. Earlier studies regarding the histopathology of NPSLE reported that microthrombi, microinfarction, and microbleeds were generally observed in the brain [20–22]. Recently, Cohen et al. reported that diffuse vasculopathy and microthrombi, which were associated with C4d and C5b-9 deposition, were uniquely observed in the brains of NPSLE patients [23]. The study suggested that the activation of the classical complement pathway plays an important role in the pathogenesis of NPSLE.

To assess the role of the complement system, the CSF concentrations of C3 and C4 have been measured in several studies of immune-mediated diseases. However, the measurement of C3 and C4 concentrations in CSF alone is not sufficient to detect the intrathecal production of complement in the CNS. Although complement components are locally produced by resident cells in the brain [24], the synthesis of complement in the brain is reported to be low or undetectable under healthy conditions [25]. Therefore, damage to the BBB allows the entry of complement from the systemic circulation resulting in high complement concentrations in the CSF. Recent studies suggest that calculation of an index (CSF concentration of complement × serum albumin/serum concentration of complement × CSF albumin), is a useful means for detecting intrathecal complement protein synthesis in the CSF in various neuropsychiatric disorders [26, 27]. Notably, in patients with NPSLE, elevated mean C3 and C4 index values were reported [28]. However, the role of complement activation in the pathogenesis of NPSLE remains unclear.

Alpha2 macroglobulin (α2MG) is a large glycoprotein found in serum with a molecular weight of 718, which inhibits many proteases including trypsin, thrombin, and collagenase. α2MG is mainly synthesized by the liver, and the CSF/serum concentration quotient (Q α2MG) has been reported to be a useful marker to evaluate BBB function [29–31]. Although Qα2MG is much less common than Q albumin, Schliep et al. reported a marked elevation of CSF α2MG levels (100 times the normal level) in patients with acute stage of purulent meningitis, blastematous meningitis, and spinal metastasis, corresponding to complete BBB disruption as evaluated by disc electrophoresis [29]. Hirohata et al. showed that Q α2MG was significantly correlated with Q albumin in patients with non-inflammatory neurological diseases (control, spinal spondylosis, cerebrovascular disease, degenerative diseases) as well as inflammatory neurological diseases (multiple sclerosis, infectious meningoencephalitis). Although there was no significant difference in the ratio of Q α2MG/Q albumin among various neurological diseases, the ratio of Q α2MG/Q albumin was significantly decreased in patients with infectious meningoencephalitis when they recovered after treatment [30].

Kanoh and Ohtani reported that the levels of CSF α2MG as well as Q α2MG in patients with viral meningitis, mycotic meningitis, and bacterial meningitis were significantly elevated compared with healthy controls and that a significant elevation of Q albumin was not observed in patients with viral meningitis, mycotic meningitis, and bacterial meningitis compared with healthy controls [31, 32].

Livrea et al. examined the relationship between the Q of proteins with different hydrodynamic radius (R) (albumin, R = 35.8 Å; IgG, R = 53.4 Å, α2MG, R = 93.5 Å) and interpreted it according to pore or vesicular BBB models. The authors suggested that heterogeneous, independent, permeability BBB mechanisms maintain the normal CSF/serum protein concentration gradient, that a set of highly selective pores with a radius less than 120 Å were responsible for the transfer of substantial albumin fraction into the CSF, and that a nonselective permeability mechanism consisting of pores and/or vesicles with a radius greater than 1000–1500 Å were responsible for the transfer of IgG and α2MG. The authors also described that a loss of selectivity was the main feature of BBB in acute meningoencephalitis, and was mediated by pores or vesicles with a radius greater than 1000–1500 Å[33]. These studies suggest that Q α2MG might be useful for the evaluation of permeability abnormalities in the BBB that cannot be evaluated by Q albumin.

In the present study, we evaluated BBB function among various subsets of NPSLE using Q α2MG instead of Q albumin. Then, we determined the levels of CSF IL-6, CSF C3, and Q C3 among the subsets of NPSLE, and evaluated the relationship between these parameters and Q α2MG. Furthermore, we calculated the C3 index using α2MG instead of albumin to determine the intrathecal synthesis of C3.

## Materials and methods

### Patients and samples

A total of 56 SLE patients who all fulfilled the American College of Rheumatology (ACR) 1982 revised criteria for the classification of SLE [34] were included in this study. Of these patients, 48 were diagnosed with NPSLE according to the 1999 ACR definition criteria of NPSLE [4] and 8 were diagnosed with non-NPSLE. Of the patients with NPSLE, 30 patients had diffuse syndromes (diffuse NPSLE) and 18 patients had focal syndromes (focal NPSLE). Diffuse NPSLE consisted of ACS (n = 14) and non-ACS diffuse NPSLE (psychosis, mood disorder, and cognitive dysfunction) (n = 16) (Table 1). Written informed consent was obtained from the patients, next of kin or legally authorized guardians. This study, including the process of securing informed consent, was approved by the ethics Committee of Fukushima Medical University (approval number 613), which is guided by local policy, national law, and the World Medical Association Declaration of Helsinki.

**Table 1:** Patient profiles.

Diagnosis	Patients (n)	Gender (male/female)	Age (mean ± SD)
**NPSLE**^a^	48	6 / 42	37.6 ± 13.2
**Diffuse NPSLE**	30	3 / 27	34.9 ± 15.2
**Acute confusional state (ACS)**	14	3 / 11	36.4 ± 16.1
**Non-ACS**	16	0 / 16	33.7 ± 12.1
**Psychosis**	10	0 / 10	28.2 ± 14.3
**Mood disorder**	5	0 / 5	44.4 ± 11.9
**Cognitive disorder**	1	0 / 1	35
**Focal NPSLE**	18	3 / 15	41.9 ± 15.7
**Non-NPSLE**	8	1 / 7	27.5 ± 5.5

^a^ NPSLE, neuropsychiatric systemic lupus erythematosus

After obtaining informed consent, CSF samples were obtained from all patients by lumbar puncture and serum samples were obtained at the same time. When CSF samples were collected, routine CSF analysis (cell count, total protein, glucose) were performed in some patients. Serum and CSF samples were kept frozen at −80°C until use.

### Detection of α2MG in CSF by western blotting

α2MG in CSF was detected by western blotting. Each CSF sample was diluted 1:10 in sample buffer, and run (1 μl/lane) on 7.5% polyacrylamide gel (SuperSep^™^ Ace, 7.5%; Wako, Osaka, Japan) under non-reducing conditions. For the positive control, 10 ng/lane of α2MG (Sigma, St. Louis, MO) was used instead of a CSF sample. The separated protein was transferred (200 mA, 25 min) to a nitrocellulose membrane (Bio-Rad Laboratories, Hercules, CA), blocked in 1% bovine serum albumin (BSA) overnight, and incubated with goat anti-human α2MG (1:1,000; Cappel, Aurora, OH). After overnight incubation at 4°C, the membrane was washed with PBS-T, and then incubated for 60 min with horseradish peroxidase (HRP)-conjugated anti-goat IgG (1:5,000; Jackson Laboratories, West Grove, PA). After washing, the membrane was developed using a SuperSignal West Dura Extended Duration Substrate kit (Thermo Scientific).

### Measurement of α2MG, IL-6, and C3

α2MG levels in CSF and sera were measured in triplicate using an enzyme-linked immunosorbent assay (ELISA). ELISA plates (96-well plates) were coated with 2 μg/ml of goat anti-human α2MG in TBS at 4°C overnight (100 μl/well). After 5 washes with TBS-T (TBS containing 0.05% Tween 20), the plates were blocked with 0.1% BlockAce^™^ (DS Pharma Biomedical Co., Osaka, Japan) at room temperature (RT) for 2 h. Purified human α2MG was used as the standard. After 5 washes with TBS-T, 100 μl of diluted samples (sera: 1:100,000 in TBS-T; CSF: 1:200 in TBS-T) were added to each well, and incubated at 4°C overnight. After 5 washes with TBS-T, the plate was incubated with HRP-conjugated anti-α2MG antibody (100 μl/well, 2.0 μg/ml; GeneTex, Irvine, CA) for 2 h. After additional washing with TBS-T, TMB substrate (Cappel) was added. To stop the color development, 1 N HCl was added, and the optical density (OD) at 450 nm was determined using a microplate reader (Benchmark Plus^™^ Microplate Spectrophotometer, Bio-Rad Laboratories). A standard curve was prepared using α2MG (3.125, 6.25, 12.5, 15, and 50 ng/ml) to calculate α2MG concentrations.

CSF IL-6 levels and C3 levels in CSF and sera were measured in triplicate using human IL-6 ELISA Ready-SET-Go!^®^ kit (eBioscience, San Diego, CA) and human C3 ELISA kit (Abcam, Cambridge, MA), respectively, according to the manufacturer’s instructions.

### Calculation of quotient alpha2 macroglobulin, quotient complement component 3, and C3 index

Quotient α2MG was calculated using the following formula: [CSF α2MG (mg/ml)/serum α2MG (mg/ml) × 1,000]. Quotient C3 was calculated using the following formula: [CSF C3 (mg/ml)/serum C3 (mg/ml) × 1,000]. C3 index was calculated using the following formula: [CSF C3 × serum α2MG/serum C3 × CSF α2MG].

### Statistical analysis

Statistical analysis was performed using Mann-Whitney’s *U*-test for the comparison of median values. Correlation coefficients were determined by Spearman’s rank correlation coefficients. Statistical analyses were performed using SPSS software, version 22 (IBM SPSS, Armonk, NY). *P* values less than 0.05 were considered statistically significant.

## Results

### Routine cerebrospinal fluid analysis in NPSLE and non-NPSLE

**Fig 1:**
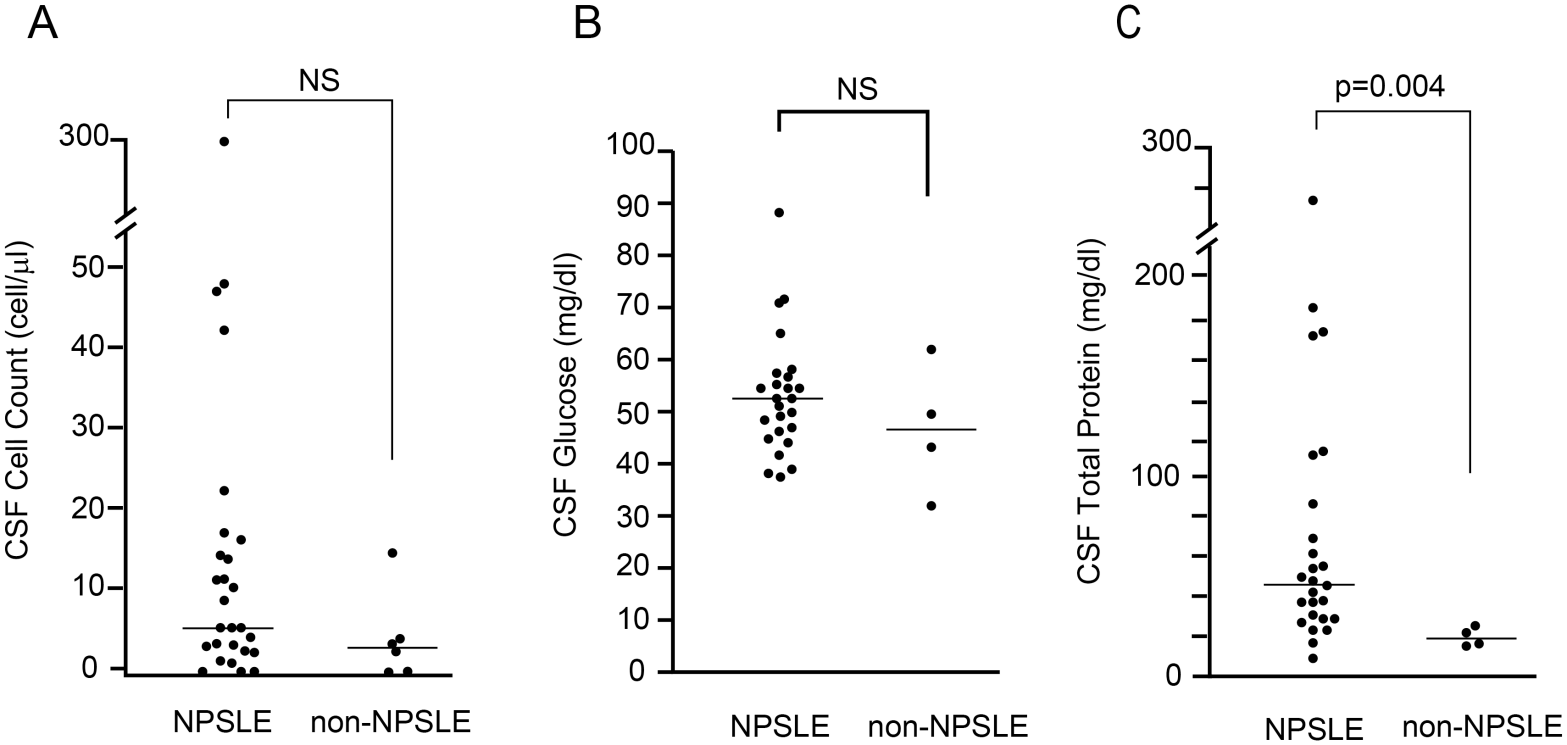
Routine cerebrospinal fluid analysis in neuropsychiatric systemic lupus erythematosus. (A) Comparison of cerebrospinal fluid (CSF) cell count between neuropsychiatric systemic lupus erythematosus (NPSLE) and non-NPSLE. (B) Comparison of CSF glucose level between NPSLE and non-NPSLE. (C) Comparison of CSF TP level between NPSLE and non-NPSLE. Horizontal lines indicate the median. Statistical analysis was performed using the Mann-Whitney *U*-test.

CSF cell count was performed in 27 NPSLE and 6 non-NPSLE. There was no significant difference in CSF cell count between NPSLE and non-NPSLE. Glucose levels in CSF were measured in 25 NPSLE and 4 non-NPSLE. There was no significant difference in CSF glucose level between NPSLE and non-NPSLE. TP levels in CSF were measured in 26 NPSLE and 4 non-NPSLE. A significant elevation of TP levels in CSF was observed in NPSLE (median 49.0 mg/dL, n = 26) compared with non-NPSLE (median 20.5 mg/dL, n = 4) (*p* = 0.004) (Fig 1).

### Detection of cerebrospinal fluid α2MG in NPSLE and non-NPSLE

**Fig 2:**
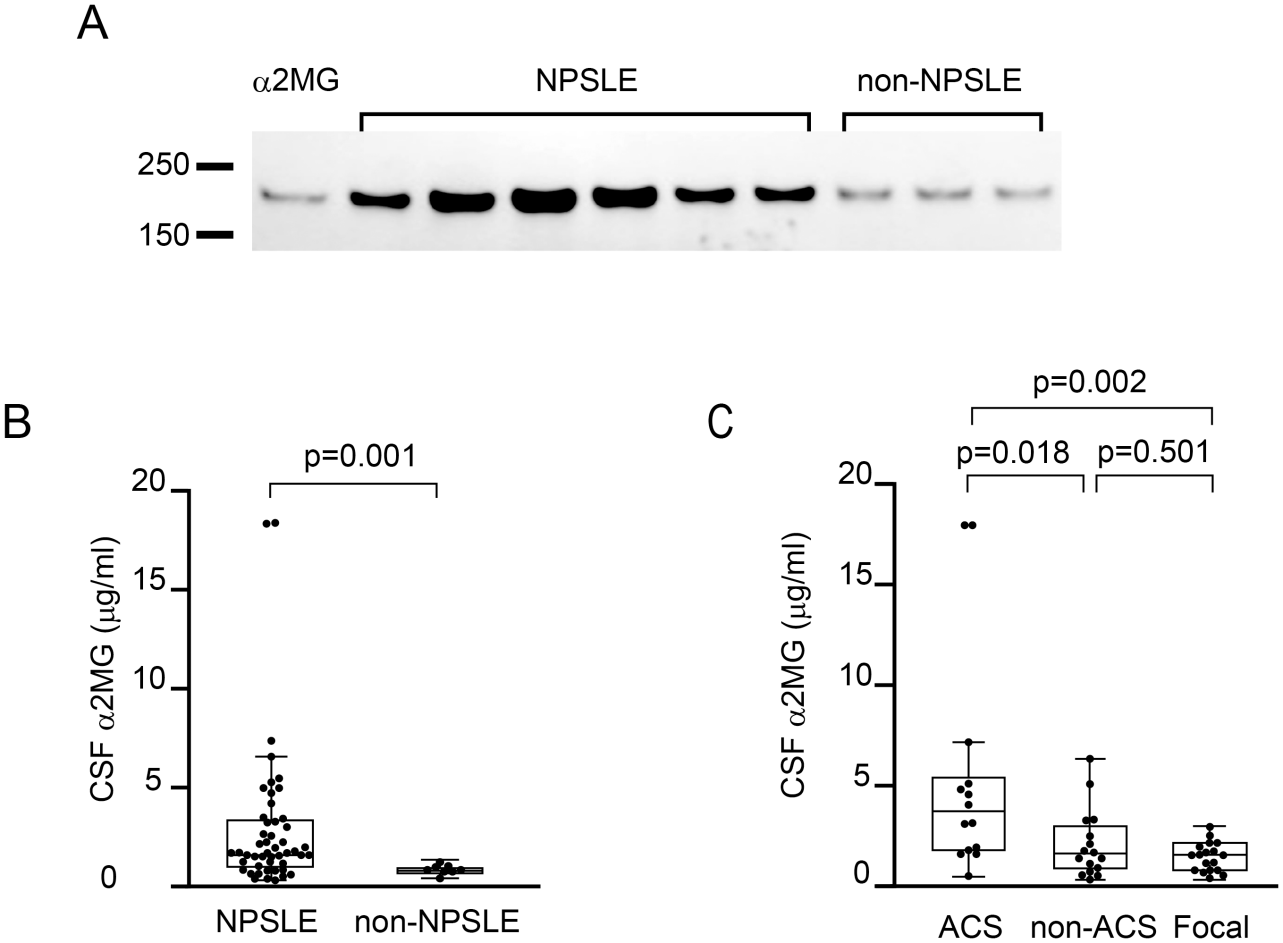
Cerebrospinal fluid alpha2 macroglobulin in patients with neuropsychiatric systemic lupus erythematosus. (A) Western blot analysis of alpha2 macroglobulin (α2MG) in cerebrospinal fluid (CSF). Upregulation of α2MG in CSF was evident in neuropsychiatric systemic lupus erythematosus (NPSLE) patients. (B) Comparison of CSF α2MG level between NPSLE and non-NPSLE. (C) Comparison of CSF α2MG level among the subsets of NPSLE. ACS, acute confusional state; non-ACS, diffuse NPSLE other than ACS; Focal, focal NPSLE. In (B) and (C), data are shown as box plots. The boxes indicate the upper and lower interquartile range (IQR), the lines within the boxes indicate the median, the whiskers indicate the minimum and maximum IQR. Each dot represents an individual sample. Statistical analysis was performed using the Mann-Whitney *U*-test.

Immunoblotting analysis using anti-α2MG antibody revealed the upregulation of α2MG in the CSF of NPSLE patients (Fig 2A). Concentrations of α2MG in the CSF of NPSLE patients (median 1.71 μg/mL, interquartile range [IQR] 1.07–3.37; n = 48) were significantly higher than those of non-NPSLE patients (median 0.72 μg/mL, IQR 0.55–0.92; n = 8) (*p* = 0.001) (Fig 2B).

Then we examined CSF α2MG levels in various subsets of NPSLE. NPSLE consists of diffuse NPSLE, including ACS and non-ACS diffuse NPSLE (psychosis, mood disorder, and cognitive dysfunction), and focal NPSLE. CSF α2MG levels were significantly increased in ACS diffuse NPSLE (median 3.81 μg/mL, IQR 1.71–5.79; n = 14) compared with non-ACS diffuse NPSLE (median 1.47 μg/mL, IQR 0.96–3.20; n = 16) (*p* = 0.018) and focal NPSLE (median 1.57 μg/mL, IQR 0.88–2.08; n = 18) (*p* = 0.002). In contrast, there was no significant difference in CSF α2MG level between non-ACS diffuse NPSLE and focal NPSLE (Fig 2C).

There were no significant differences in serum α2MG levels between NPSLE and non-NPSLE, as well as among the subsets of NPSLE (data not shown).

### Evaluation of blood–brain barrier function by quotient of α2MG in NPSLE

**Fig 3:**
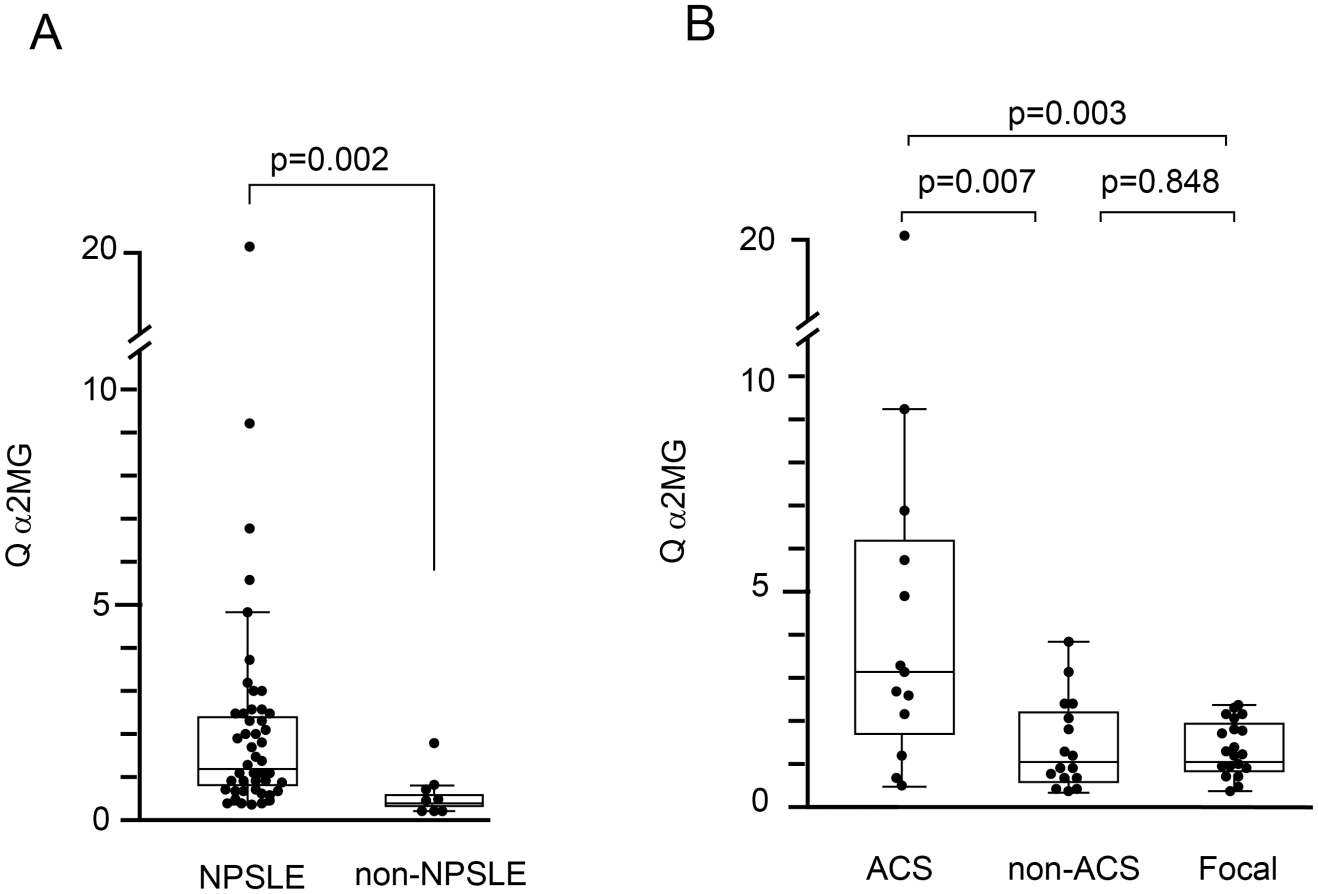
Quotient of alpha2 macroglobulin in patients with neuropsychiatric systemic lupus erythematosus. (A) Comparison of quotient alpha2 macroglobulin (Q α2MG) level between neuropsychiatric systemic lupus erythematosus (NPSLE) and non-NPSLE. (B) Comparison of Q α2MG among subsets of NPSLE. ACS, acute confusional state; non-ACS, diffuse NPSLE other than ACS; Focal, focal NPSLE. Data are shown as box plots. The boxes indicate the upper and lower interquartile range (IQR), the lines within the boxes indicate the median, the whiskers indicate the minimum and maximum IQR Each dot represents an individual sample. Statistical analysis was performed using the Mann-Whitney *U*-test.

We next determined Q α2MG values, which reflect BBB function, in 47 NPSLE and 8 non-NPSLE patients. In 1 patient with ACS diffuse NPSLE, the Q α2MG value was not determined. As shown in Fig 3A, the Q α2MG in NPSLE patients (median 1.23, IQR 0.79–2.44; n = 47) was significantly higher than that of non-NPSLE patients (median 0.42, IQR 0.23–0.73; n = 8) (*p* = 0.002).

Among the subsets of NPSLE, Q α2MG values were significantly increased in ACS diffuse NPSLE (median 3.03, IQR 1.70–6.26; n = 13) compared with non-ACS diffuse NPSLE (median 1.04, IQR 0.56–2.28; n = 16) (*p* = 0.007) and focal NPSLE (median 1.09, IQR 0.81–1.99; n = 18) (*p* = 0.003). In contrast, there was no significant difference in Q α2MG between non-ACS diffuse NPSLE and focal NPSLE (Fig 3B). These data suggest that BBB damage is present in patients with NPSLE, especially in ACS diffuse NPSLE.

### Cerebrospinal fluid IL-6 levels in NPSLE and non-NPSLE

**Fig 4:**
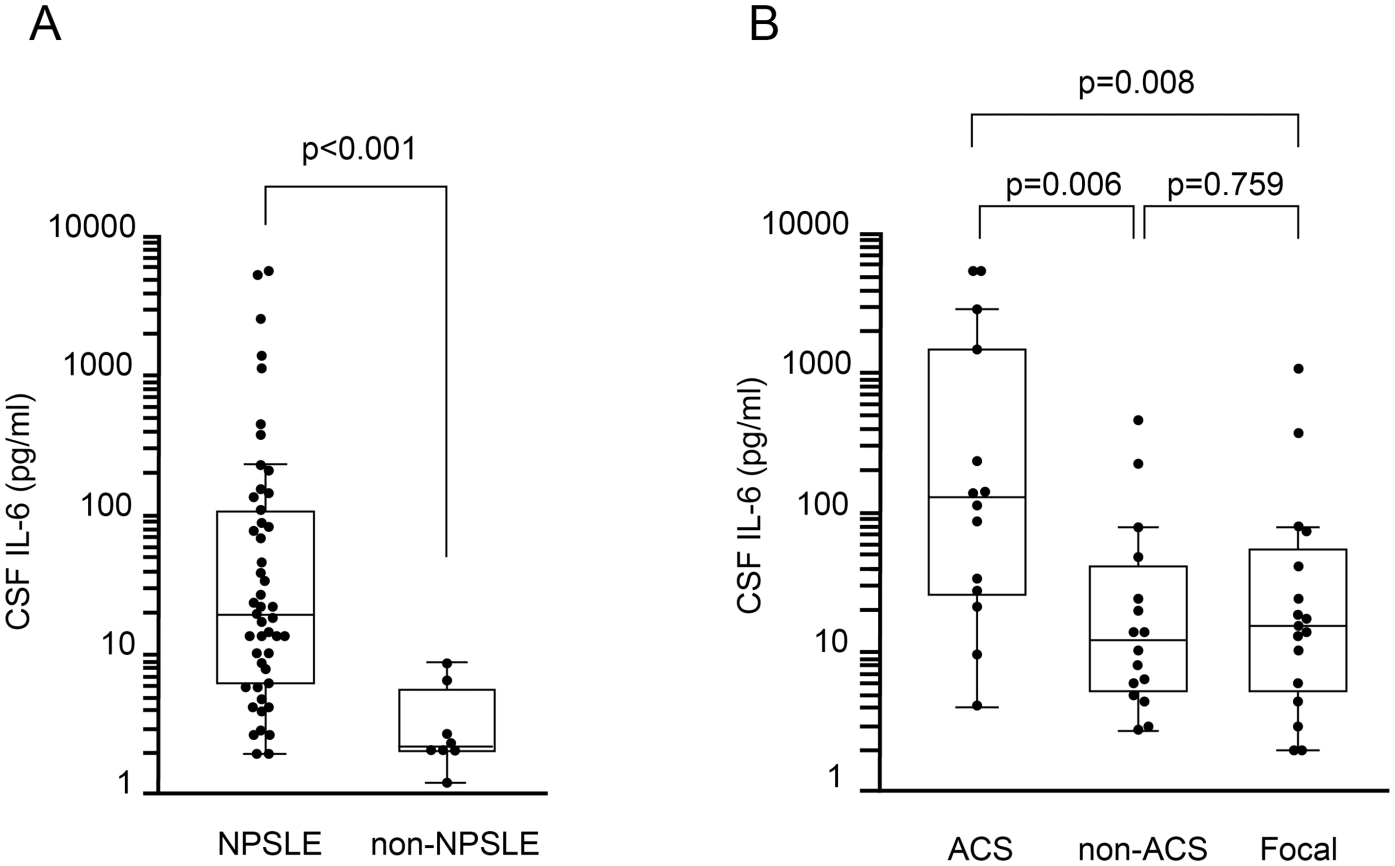
Cerebrospinal fluid interleukin-6 levels in patients with neuropsychiatric systemic lupus erythematosus. (A) Comparison of cerebrospinal fluid (CSF) interleukin (IL)-6 level between neuropsychiatric systemic lupus erythematosus (NPSLE) and non-NPSLE. (B) Comparison of CSF IL-6 level among subsets of NPSLE. ACS, acute confusional state; non-ACS, diffuse NPSLE other than ACS; Focal, focal NPSLE. Data are shown as box plots. The boxes indicate the upper and lower interquartile range (IQR), the lines within the boxes indicate the median, the whiskers indicate the minimum and maximum IQR Each dot represents an individual sample. Statistical analysis was performed using the Mann-Whitney *U*-test.

We determined IL-6 levels in the CSF of 47 NPSLE and 8 non-NPSLE patients. In 1 patient with focal NPSLE, CSF IL-6 was not determined. As shown in Fig 4A, IL-6 concentration in the CSF of NPSLE patients (median 19.60 pg/mL, IQR 6.60–115.80; n = 47) was significantly higher than that of non-NPSLE patients (median 2.15 pg/mL, IQR 2.00–5.45; n = 8) (*p* < 0.001).

Next, we examined the IL-6 levels in various subsets of NPSLE. IL-6 concentration was significantly increased in CSF in ACS diffuse NPSLE (median 124.30 pg/ml, IQR 26.20–1772.82; n = 14) compared with non-ACS diffuse NPSLE (median 12.00 pg/ml, IQR 5.34–41.88; n = 16) (*p* = 0.006) and focal NPSLE (median 15.60 pg/ml, IQR 5.20–56.60; n = 17) (*p* = 0.008). In contrast, there was no significant difference in CSF IL-6 concentration between non-ACS diffuse NPSLE and focal NPSLE (Fig 4B).

### Relationship between blood–brain barrier integrity and the cell count, total protein level, and glucose level in cerebrospinal fluid from NPSLE patients

There was no significant correlation between CSF cell count and Q α2MG, or between CSF glucose level and Q α2MG in NPSLE patients.

**Fig 5:**
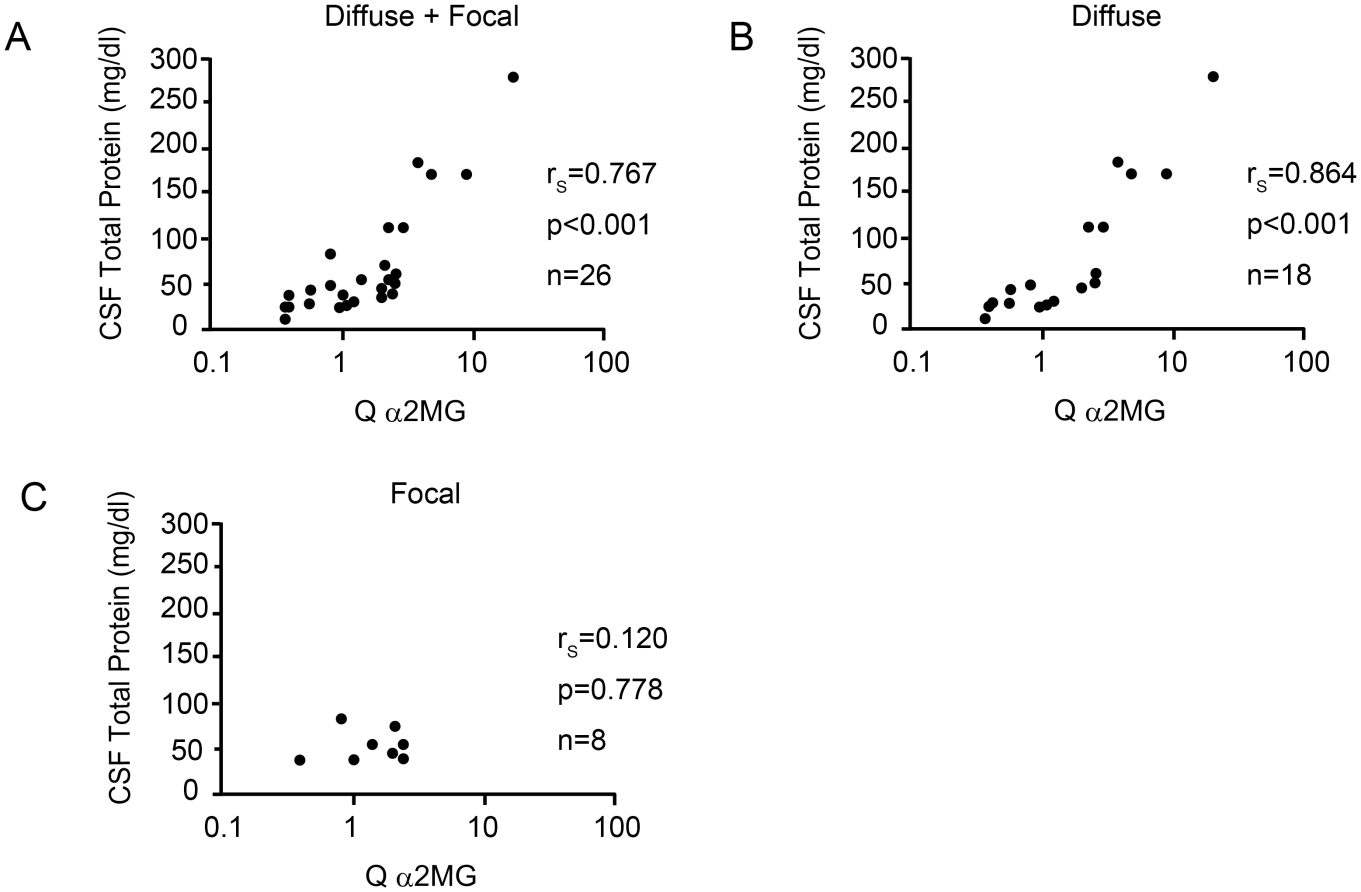
Correlation between cerebrospinal fluid total protein level and the quotient of alpha2 macroglobulin in patients with neuropsychiatric systemic lupus erythematosus. (A) Correlation between cerebrospinal fluid (CSF) total protein (TP) level and quotient of alpha2 macroglobulin (Q α2MG) in all neuropsychiatric systemic lupus erythematosus (NPSLE) patients. (B) Correlation between CSF TP and Q α2MG in diffuse NPSLE patients. (C) Correlation between CSF TP and Q α2MG in focal NPSLE patients. Statistical significance was determined using the Spearman’s rank correlation test.

However, CSF TP level was significantly correlated with Q α2MG in NPSLE patients (r_s_ = 0.767, *p* < 0.001). Furthermore, among the subsets of NPSLE, CSF TP level was also significantly correlated with Q α2MG in diffuse NPSLE (r_s_ = 0.864, *p* < 0.001). In contrast, there was no significant correlation between CSF TP level and Q α2MG in focal NPSLE (r_s_ = 0.120, *p* = 0.778) (Fig 5).

### Relationship between blood–brain barrier integrity and cerebrospinal fluid IL-6 levels in NPSLE

**Fig 6:**
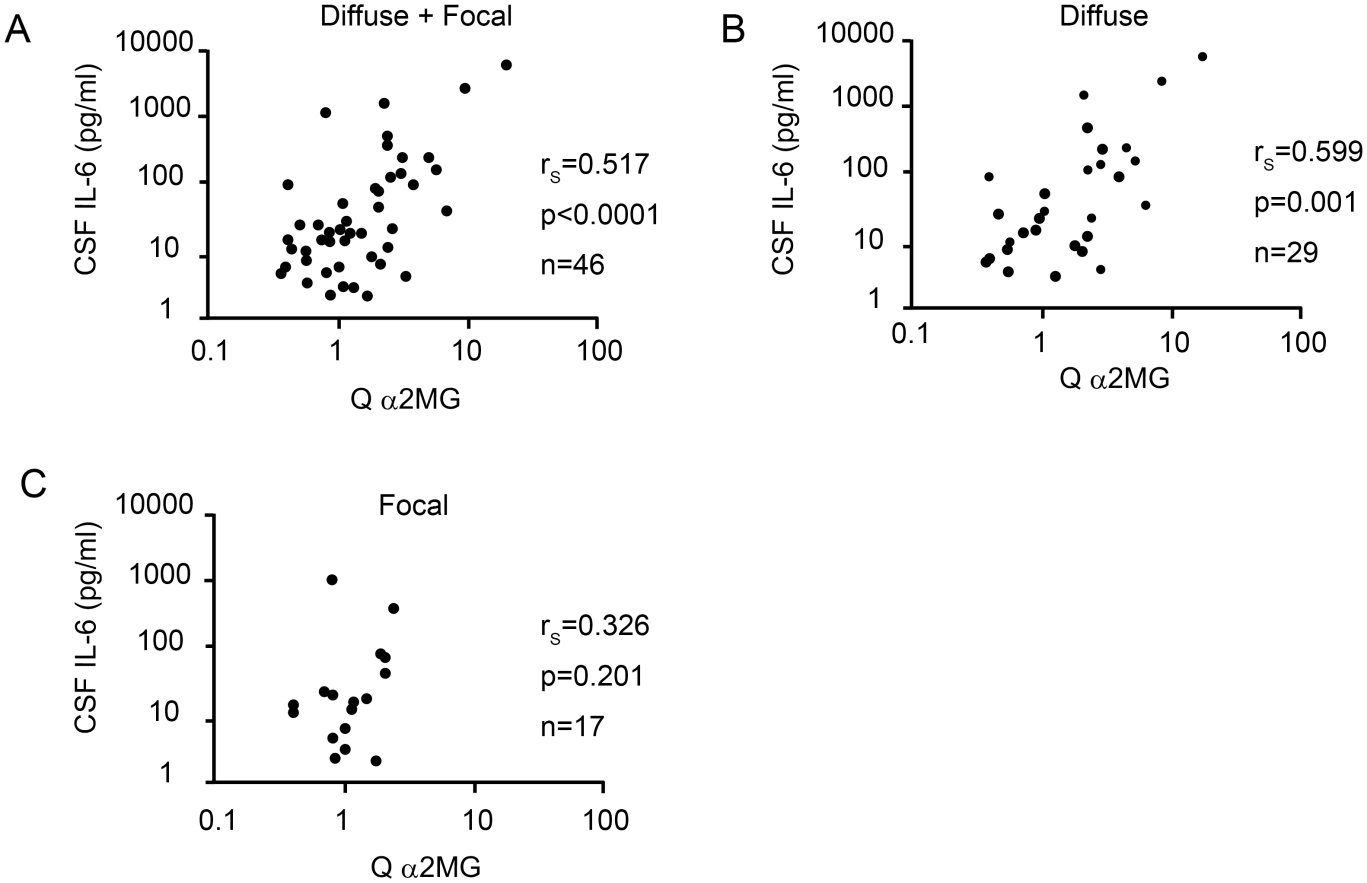
Correlation between cerebrospinal fluid interleukin-6 concentration and the quotient of alpha2 macroglobulin in patients with neuropsychiatric systemic lupus erythematosus. (A) Correlation between cerebrospinal fluid (CSF) interleukin (IL)-6 concentration and quotient of alpha2 macroglobulin (Q α2MG) in all neuropsychiatric systemic lupus erythematosus (NPSLE) patients. (B) Correlation between CSF IL-6 and Q α2MG in diffuse NPSLE patients. (C) Correlation between CSF IL-6 and Q α2MG in focal NPSLE patients. Statistical significance was determined using Spearman’s rank correlation test.

CSF IL-6 level was significantly correlated with Q α2MG in NPSLE patients (r_s_ = 0.517, *p* < 0.0001) (Fig 6A). Furthermore, among the subsets of NPSLE, CSF IL-6 level was also significantly correlated with Q α2MG in diffuse NPSLE (r_s_ = 0.599, *p* = 0.001) (Fig 6B). In contrast, there was no significant correlation between CSF IL-6 level and Q α2MG in focal NPSLE (r_s_ = 0.326, *p* = 0.201) (Fig 6C).

### Cerebrospinal fluid and serum C3 levels in NPSLE and non-NPSLE

**Fig 7:**
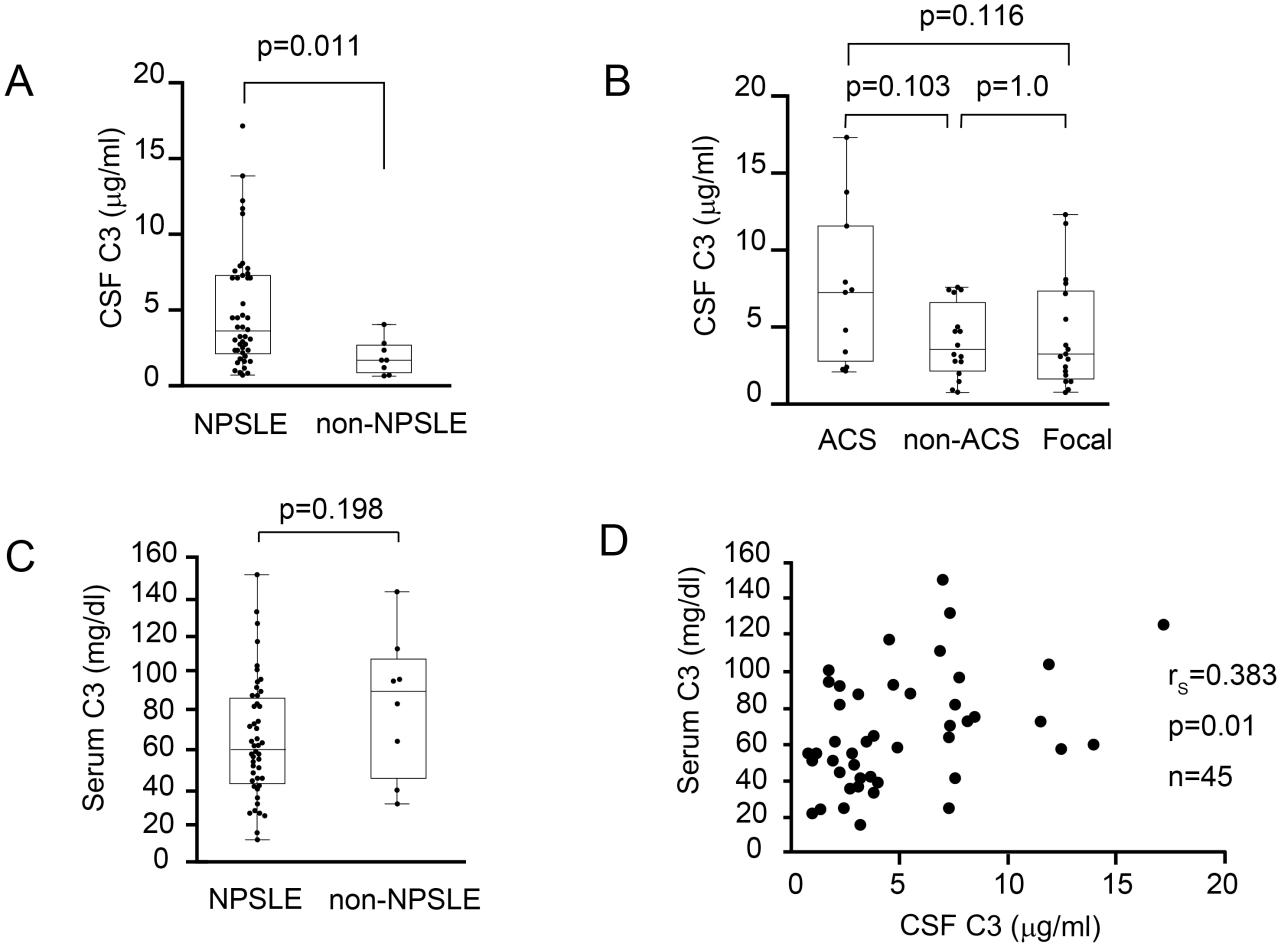
Complement component 3 levels in cerebrospinal fluid and serum. (A) Comparison of cerebrospinal fluid (CSF) and complement component 3 (C3) level between neuropsychiatric systemic lupus erythematosus (NPSLE) and non-NPSLE. (B) Comparison of CSF C3 level among the subsets of NPSLE. ACS, acute confusional state; non-ACS, diffuse NPSLE other than ACS; Focal, focal NPSLE. (C) Comparison of serum C3 level between NPSLE and non-NPSLE. (D) Correlation between serum C3 level and CSF C3 level. In (A), (B), and (C), data are shown as box plots. The boxes indicate the upper and lower interquartile range (IQR), the lines within the boxes indicate the median, the whiskers indicate the minimum and maximum IQR. Each dot represents an individual sample. Statistical analysis was performed using the Mann-Whitney *U*-test. In (D), statistical analysis was performed using Spearman’s rank correlation test.

We determined C3 concentration in the CSF of 45 NPSLE and 8 non-NPSLE patients. In 3 patients with ACS diffuse NPSLE, CSF C3 concentration was not determined. CSF C3 concentration in NPSLE patients (median 3.68 μg/mL, IQR 2.20–7.21; n = 45) was significantly higher than that in non-NPSLE patients (median 1.75 μg/mL, IQR 0.86–2.78; n = 8) (*p* = 0.011) (Fig 7A). There were no statistically significant differences in CSF C3 concentration among the subsets of NPSLE (ACS diffuse NPSLE: median 7.18 μg/ml, IQR 2.24–11.53; n = 11; non-ACS diffuse NPSLE: median 3.48 μg/ml, IQR 2.08–6.55; n = 16; focal NPSLE: median 3.23 μg/ml, IQR 1.74–7.29) (Fig 7B).

Next, we determined the serum C3 level in 48 NPSLE and 8 non-NPSLE patients. There was no significant difference in serum C3 level between NPSLE patients and non-NPSLE patients (Fig 7C). However, CSF C3 level was significantly correlated with serum C3 level in NPSLE patients (r_s_ = 0.383, *p* = 0.01), although the correlation was weak (Fig 7D).

### Quotient of C3 in NPSLE and non-NPSLE

**Fig 8:**
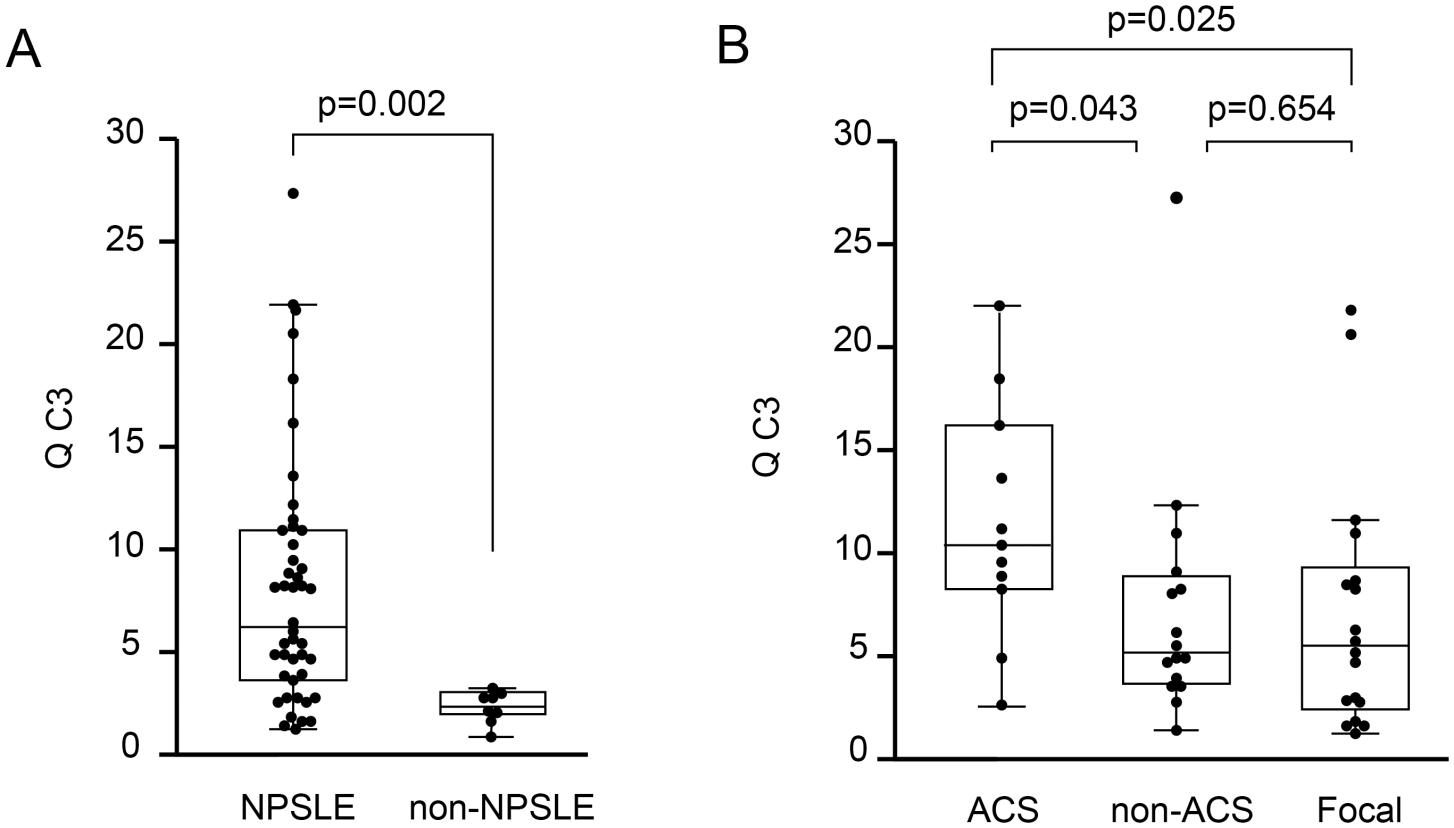
Quotient of complement component 3 in patients with neuropsychiatric systemic lupus erythematosus. (A) Comparison of quotient of complement component 3 (Q C3) value between neuropsychiatric systemic lupus erythematosus (NPSLE) and non-NPSLE. (B) Comparison of Q C3 value among the subsets of NPSLE. ACS, acute confusional state; non-ACS, diffuse NPSLE other than ACS; Focal, focal NPSLE. Data are shown as box plots. The boxes indicate the upper and lower interquartile range (IQR), the lines within the boxes indicate the median, the whiskers indicate the minimum and maximum IQR. Each dot represents an individual sample. Statistical analysis was performed by the Mann-Whitney *U*-test.

We next examined Q C3 values in 45 NPSLE and 8 non-NPSLE patients. In 3 patients with ACS diffuse NPSLE, Q C3 was not determined. As shown in Fig 8A, Q C3 level in NPSLE patients (median 6.20, IQR 3.61–11.06; n = 45) was significantly higher than that in non-NPSLE patients (median 2.44, IQR 1.91–3.01; n = 8) (*p* = 0.002).

Then, we determined Q C3 values in various subsets of NPSLE. Q C3 values were significantly increased in ACS diffuse NPSLE (median 10.25, IQR 8.10–16.03; n = 11) compared with non-ACS diffuse NPSLE (median 5.19, IQR 3.69–8.82; n = 16) (*p* = 0.043) and focal NPSLE (median 5.61, IQR 2.31–9.18; n = 18) (*p* = 0.025). In contrast, there was no significant difference in Q C3 value between non-ACS diffuse NPSLE and focal NPSLE (Fig 8B).

### Relationship between blood–brain barrier integrity and quotient of C3 in NPSLE

**Fig 9:**
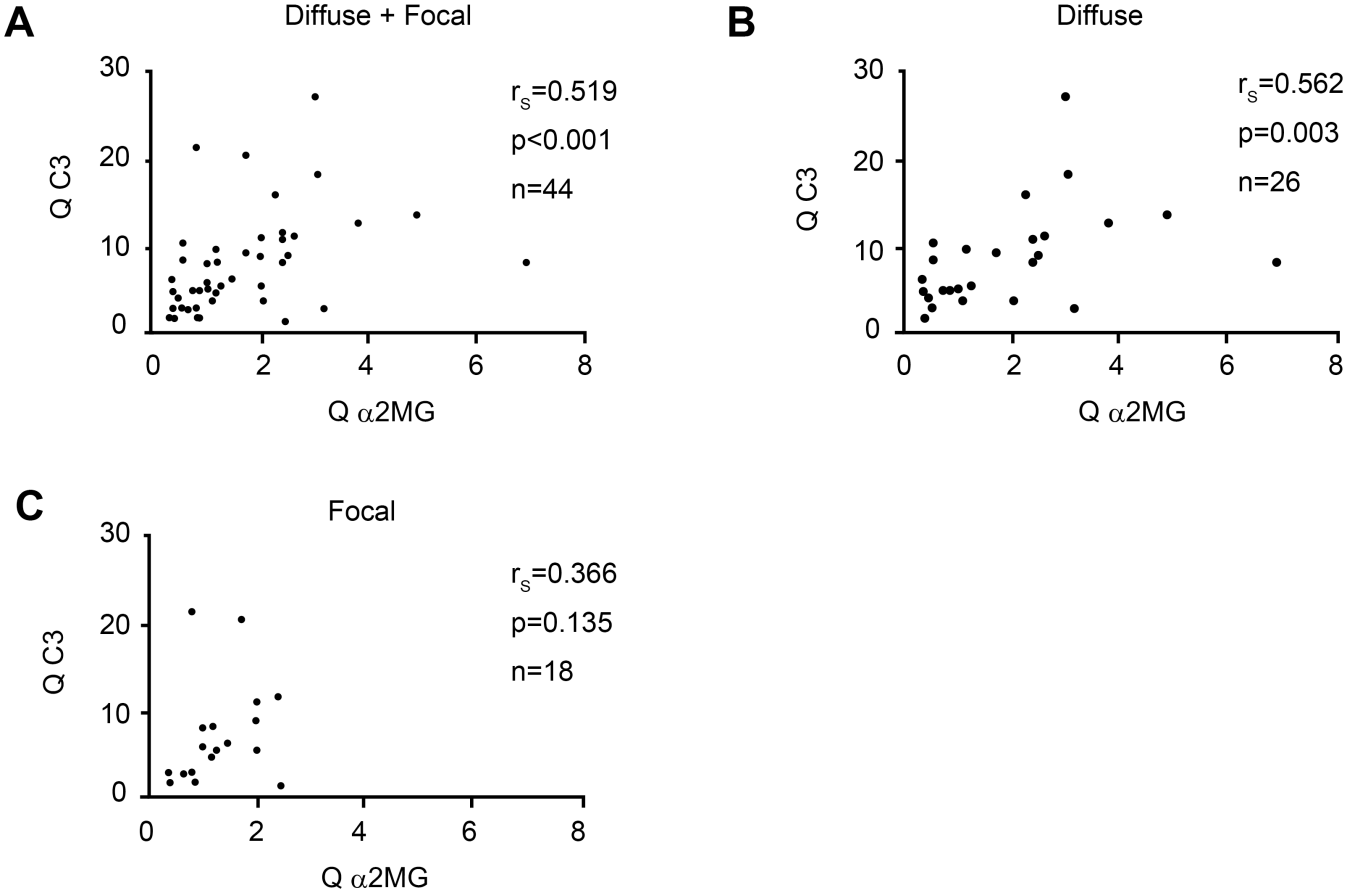
Correlation between the quotient of complement component 3 (Q C3) and the quotient of alpha2 macroglobulin (Q α2MG) in patients with neuropsychiatric systemic lupus erythematosus. (A) Correlation between Q C3 and Q α2MG in all neuropsychiatric systemic lupus erythematosus (NPSLE) patients. (B) Correlation between Q C3 and Q α2MG in diffuse NPSLE patients. (C) Correlation between Q C3 and Q α2MG in focal NPSLE patients. Statistical significance was determined using Spearman’s rank correlation test.

Q C3 was significantly correlated with Q α2MG in NPSLE patients (r = 0.519, *p* < 0.001) (Fig 9A). Among the subsets of NPSLE, Q C3 was significantly correlated with Q α2MG in diffuse NPSLE (r_s_ = 0.562, *p* = 0.003) (Fig 9B). In contrast, there was no significant correlation between Q C3 level and Q α2MG in focal NPSLE (r_s_ = 0.368, *p* = 0.135) (Fig 9C).

### C3 index in NPSLE

**Fig 10:**
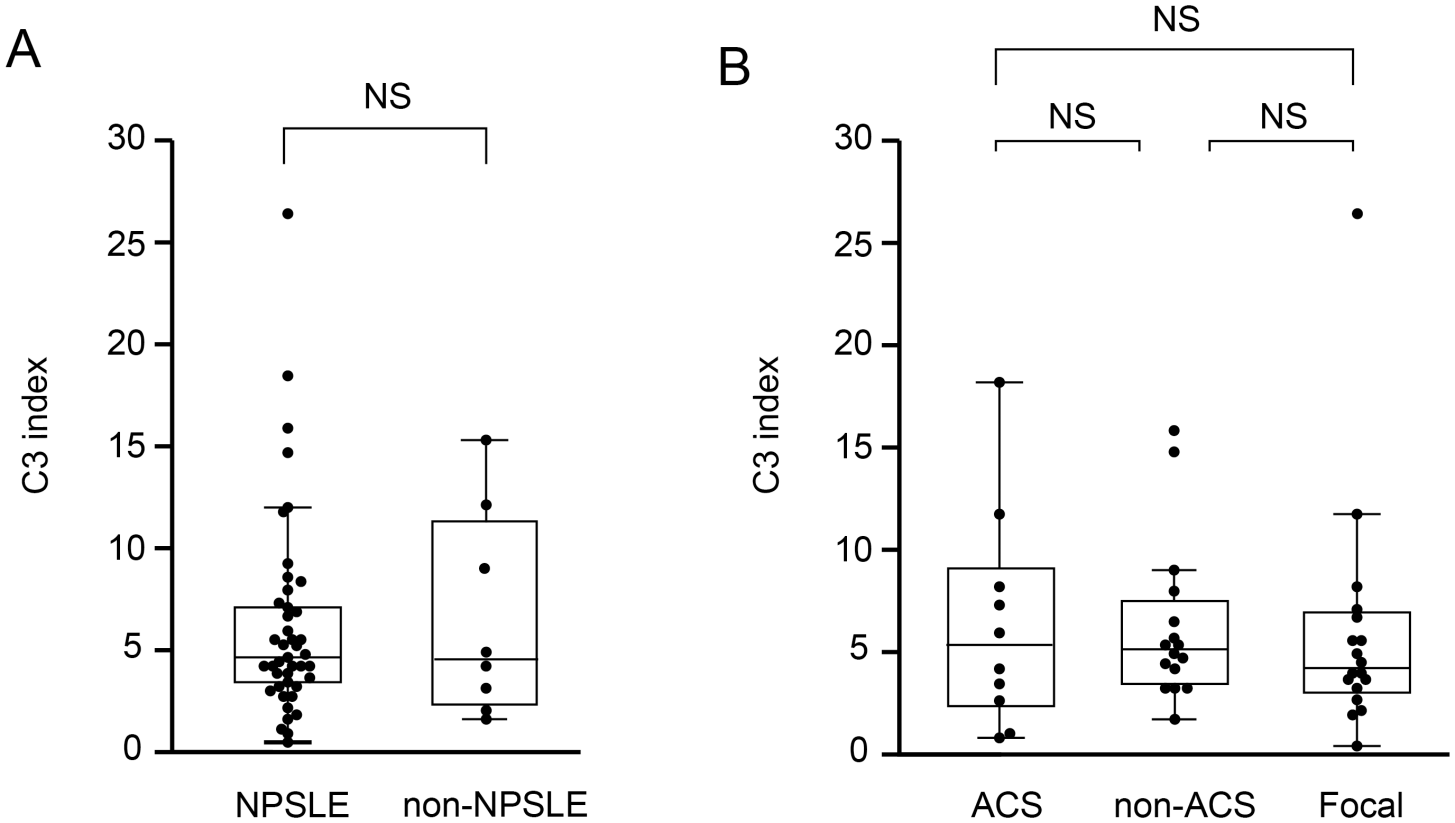
Complement component 3 (C3) index (quotient of complement component 3 (Q C3)/quotient of alpha2 macroglobulin (Q α2MG)). (A) Comparison of C3 index between NPSLE and non-NPSLE. (B) Comparison of C3 index among the subsets of NPSLE. ACS, acute confusional state; non-ACS, diffuse NPSLE other than ACS; Focal, focal NPSLE. Data are shown as box plots. The boxes indicate the upper and lower interquartile range (IQR), the lines within the boxes indicate the median, the whiskers indicate the minimum and maximum IQR. Each dot represents an individual sample. Statistical analysis was performed using the Mann-Whitney *U*-test.

To examine intrathecal C3 production in NPSLE, we determined the C3 index in 44 NPSLE and 8 non-NPSLE patients. In 4 patients with ACS diffuse NPSLE, C3 index value was not determined. There was no significant difference in C3 index value between NPSLE and non-NPSLE (Fig 10A), or between subsets of NPSLE (Fig 10B).

## Discussion

Our study had three major findings. First, Q α2MG was significantly elevated in NPSLE compared with non-NPSLE. Among the subsets of NPSLE, a significant increase of Q α2MG was also observed in ACS diffuse NPSLE. These results suggest that BBB disruption is present in patients with NPSLE, especially in ACS diffuse NPSLE.

Second, CSF IL-6 concentration was significantly elevated in NPSLE compared with non-NPSLE. Among the subsets of NPSLE, CSF IL-6 level was also increased in ACS diffuse NPSLE compared with non-ACS diffuse NPSLE or focal NPSLE. A positive correlation between CSF IL-6 concentration and Q α2MG in NPSLE was determined. Among the subgroups of NPSLE, a positive correlation was also observed in diffuse NPSLE. In contrast, there was no correlation in focal NPSLE. These results suggest that increased IL-6 in the CSF in diffuse NPSLE (especially in ACS diffuse NPSLE) is not only a result of intrathecal synthesis but also a result of transfer of IL-6 from the systemic circulation due to a damaged BBB.

Third, we observed a significant elevation of CSF C3 concentration and Q C3 value in NPSLE compared with non-NPSLE. Furthermore, CSF C3 concentration and Q C3 were also increased in ACS diffuse NPSLE compared with non-ACS diffuse NPSLE and focal NPSLE. A positive correlation between Q C3 and Q α2MG was determined, especially in diffuse NPSLE. There was no significant difference in C3 index between NPSLE and non-NPSLE, as well as among the subgroups of NPSLE. These results suggest that the source of C3 in the CSF is mainly a result of the transfer of C3 from the systemic circulation due to BBB disruption, especially in diffuse NPSLE.

In NPSLE, mild abnormalities in routine CSF analysis commonly include pleocytosis (22–36%), elevated TP level (30–66%), and low glucose level (8–42%) [35]. In this study, we observed elevated CSF TP level in NPSLE compared with non-NPSLE, which is consistent with past reports. However, the number of patients, especially non-NPSLE, is too small to draw definitive conclusions. Further investigation with larger sample sizes is needed.

Furthermore, we observed a strong correlation between CSF TP level and Q α2MG in diffuse NPSLE, especially in ACS diffuse NPSLE. Albumin forms the majority of CSF TP but is not produced in the brain; therefore, our result may support the possibility that BBB disruption is present in diffuse NPSLE, especially in ACS diffuse NPSLE.

In NPSLE, two main mechanisms have been implicated in BBB disruption. One is microthrombi in cerebral vessels resulting in ischemia, and the other is immune-mediated endothelial activation resulting in intrathecal cytokine synthesis [11]. Presently, Q albumin is the most reliable and commonly used biomarker for BBB integrity, because albumin is produced solely in the liver. Therefore, the presence of albumin in the CSF is a result of its entry through the BBB from the systemic circulation. Several earlier studies performed an evaluation of BBB integrity in NPSLE using Q albumin. Winfield et al. reported significantly elevated Q albumin in NPSLE patients with major neurologic defects (encephalopathy with disturbance in consciousness, transverse myelopathy, paraparesis) [36]. Furthermore, Hirohata et al. reported that 4 of 12 patients with NPSLE showed elevated Q albumin [37]. Recently, Hirohata et al. also determined Q albumin in various subtypes of NPSLE, and found that Q albumin was: (1) significantly elevated in NPSLE compared with non-NPSLE; (2) significantly elevated in ACS diffuse NPSLE compared with non-ACS diffuse NPSLE or focal NPSLE; and (3) was not significantly different between non-ACS diffuse NPSLE and focal NPSLE [14]. In the present study, we evaluated the BBB integrity among the subsets of NPSLE using Q α2MG instead of Q albumin. Our results on the BBB integrity in NPSLE, especially in ACS diffuse NPSLE, were consistent with earlier studies, suggesting that Q α2MG is a useful index in addition to Q albumin to evaluate the integrity of the BBB.

Several studies have been reported that IL-6 levels are increased in CSF in patients with NPSLE. Hirohata et al. demonstrated that IL-6 activity in CSF in NPSLE patients, including diffuse NPSLE and focal NPSLE, was significantly increased compared with non-NPSLE. They also showed no significant correlation between IL-6 activity and Q albumin. Tryslerg et al. measured IL-6 levels in the CSF in 14 patients with NPSLE (four organic brain syndrome (OBS), three stroke, two transverse myelitis, one meningitis, one movement disorder) and compared them with non-NPSLE patients. They found a 10-fold increase of IL-6 in NPSLE patients, especially in four patients with OBS, consisting mainly of ACS diffuse NPSLE. Katsumata et al. divided NPSLE into ACS and non-ACS (including non-ACS diffuse NPSLE and focal NPSLE), and measured IL-6 levels in the CSF of each group. They demonstrated IL-6 levels were significantly higher in ACS than non-ACS. These findings were supported by a multicenter retrospective study in which CSF IL-6 had high sensitivity and specificity in patients with lupus psychosis [19].

Regarding the source of IL-6 in the CSF of NPSLE, earlier studies suggest that the elevation of CSF IL-6 in NPSLE seemed to be a result of intrathecal synthesis of IL-6, because enhanced expression of IL-6 mRNA is present in the hippocampus of diffuse NPSLE patients [38], and there was no significant correlation between either CSF IL-6 activity and serum IL-6 activity, or between CSF IL-6 and Q albumin [16–18]. Distinct from earlier studies, we evaluated BBB function using Q α2MG instead of Q albumin. As described above, we observed a positive correlation between CSF IL-6 and Q α2MG, which raised the possibility that IL-6 in CSF in ACS diffuse NPSLE is not only a result of intrathecal production but also a result of the entry of IL-6 from the systemic circulation to the CSF through the disrupted BBB. Considering the high values of Q albumin and Q α2MG in ACS diffuse NPSLE, which suggest BBB disruption, it is likely that IL-6 from the systemic circulation enters the CSF through the damaged BBB in patients with ACS diffuse NPSLE.

Studies previously investigated the relationship between CSF complement levels and NPSLE. Jongon et al. [28] performed a comparative study of C3 and C4 indexes (C3 index: Q C3/Q albumin, C4 index: Q C4/Q albumin) in patients with various immunological disorders including NPSLE, and found increased mean C3 and C4 index values in NPSLE, which indicated a positive correlation between the C3 index and IgG index, and between the C4 index and IgM index.

However, conflicting results were reported regarding the C3 index in NPSLE from the same authors [39]. Jongon showed no statistical difference in C3 index between NPSLE and control patients, which is consistent with our result. This discrepancy may be because of a difference in the clinical state of the patients. In the study reporting increased C3 index in NPSLE, there was no statistical difference in Q albumin between NPSLE and the control group, which means the number of NPSLE patients with BBB impairment was small.

In this study, we found no significant difference in C3 index between NPSLE and non-NPSLE, as well as among the subgroups of NPSLE. However, in SLE patients with hypocomplementemia due to high disease activity, complement index may not reflect intrathecal complement synthesis alone because low serum complement concentration may increase the quotient level, resulting in an increased index. Measuring complement cleavage products (C3d, C4d) in CSF may be more appropriate for the evaluation of the intrathecal complement activation. Thus, further study is required.

In patients with diffuse NPSLE, we observed a significant positive correlation between Q α2MG and CSF TP, Q α2MG and CSF IL-6, and Q α2MG and Q C3. The strong correlation between Q α2MG and CSF TP may be because the majority of CSF TP is albumin, which is not produced in the brain. Compared with the correlation between Q α2MG and CSF TP, the correlation between Q α2MG and CSF IL-6, as well as between Q α2MG and Q C3 were not so strong (moderate). For the correlation between Q α2MG and CSF IL-6, this might be because of the increased synthesis of IL-6 in the brain in ACS diffuse NPSLE [38], which might reduce correlation coefficient between Q α2MG and CSF IL-6. For the correlation between Q α2MG and Q C3, patients with hypocomplementemia may influence the correlation coefficient. Increased Q C3 because of low serum C3 may reduce the correlation coefficient between Q α2MG and Q C3.

## Conclusions

Our study suggested that elevated levels of IL-6 and C3 in CSF in diffuse NPSLE, especially in ACS, are the result of entry from the systemic circulation to the CSF through the damaged BBB, as well as the result of increased intrathecal production. Furthermore, it is possible that Q α2MG is a useful measure in addition to Q albumin to evaluate the integrity of the BBB.
